# First person – Komal Panchal

**DOI:** 10.1242/bio.055632

**Published:** 2020-09-03

## Abstract

First Person is a series of interviews with the first authors of a selection of papers published in Biology Open, helping early-career researchers promote themselves alongside their papers. Komal Panchal is first author on ‘Miro, a Rho GTPase genetically interacts with Alzheimer's disease-associated genes (*Tau*, *Aβ_42_* and *Appl*) in *Drosophila melanogaster*’, published in BiO. Komal is a PhD student in the lab of Dr Anand K. Tiwari at the Institute of Advanced Research (IAR), Koba Institutional Area, Gujarat, India, investigating the possible molecular basis of Alzheimer's disease.


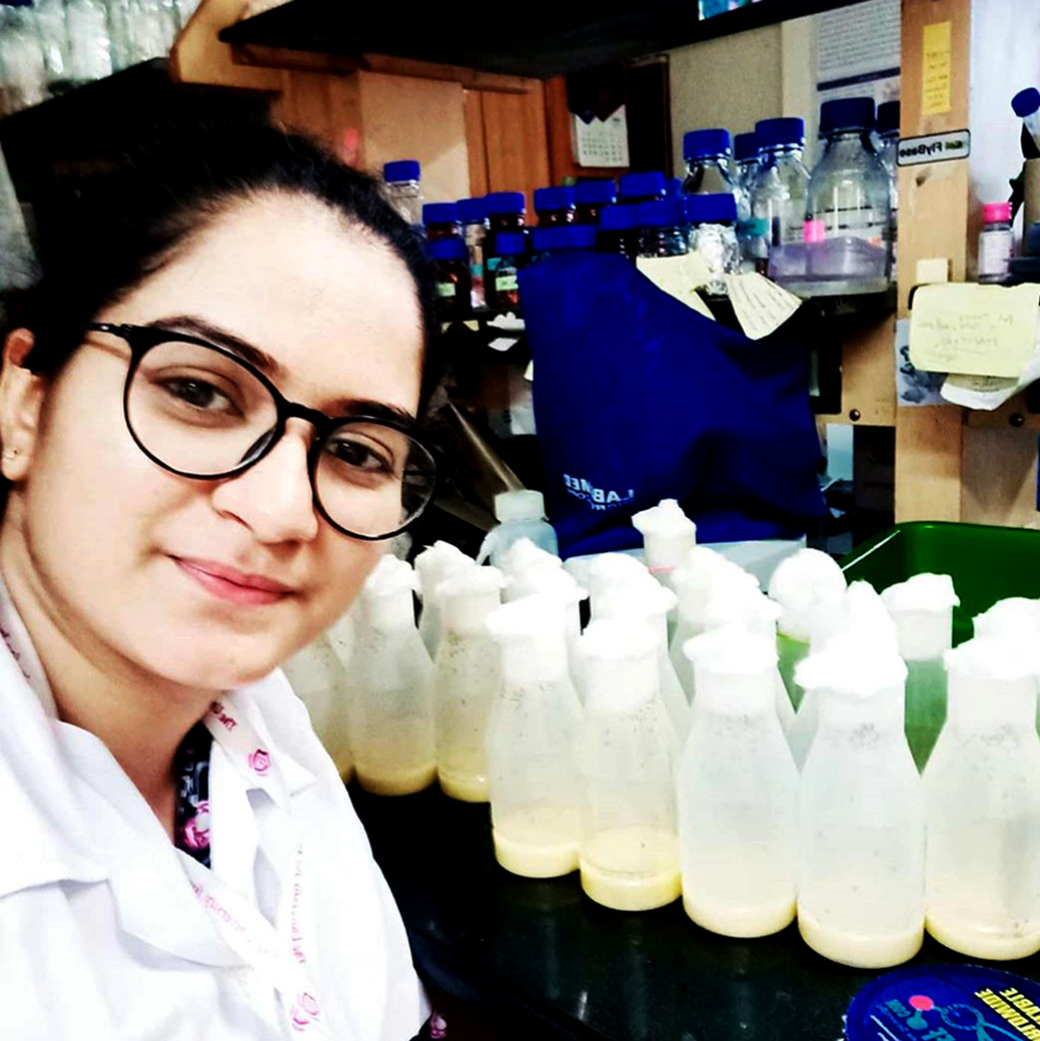


**Komal Panchal**

**What is your scientific background and the general focus of your lab?**

My scientific background is in genetics and molecular biology and my lab's focus is to identify the *in vivo* genetic modifier like *Miro* and *Sirtuins* genes in Alzheimer's disease (AD). We focused our study on how *Miro* and *Sirtuins* genes interact with AD-causing genes *Appl* and *Tau*, and modulate AD-related pathologies.

**How would you explain the main findings of your paper to non-scientific family and friends?**

My research work is based on AD, which is the most common form of dementia and most prevalent neurodegenerative disease worldwide. The common symptoms of AD include memory loss, mental confusion, depression, and speech, behavior and motor system problems. More than 54 million people are suffering from AD worldwide and this number is growing continuously. However, significant advancements in terms of science and technology have taken place, but still there are no effective cures/treatments for AD available. Thus, it is worth doing more research to find out the molecular basis of AD-related pathologies, which might be helpful to find out the most effective therapeutic targets of AD. We have used *Drosophila melanogaster*, a tiny ‘fruit fly’ to study AD. Fruit flies have a ∼75% human disease related protein similarity and possess a complex central nervous system (CNS) and mimic AD-related behavior defects. By using the *Drosophila* model of AD, we have found that Miro, a mitochondrial outer membrane protein, genetically interacts with AD-associated genes *Appl* and *Tau*, and positively modulates AD-related pathologies such as behavior defects, energy deficiency, cell death and neurodegeneration. Thus, our current study has suggested that Miro might play a key role in therapeutic intervention targeting AD-related pathologies.

**What are the potential implications of these results for your field of research?**

As mentioned above, AD is one of the most challenging problems across the globe and needs immediate attention. Although several past and current pieces of research have significantly improved our understanding about AD, the molecular details and the interacting partners of AD-associated genes are not well understood. In our study, we focused our research to mitochondrial axonal transport protein Miro and tried to explore its genetic interaction with AD-related genes in *Drosophila*. Our findings suggested that overexpression of *Miro* reduces the AD-related pathologies in *Drosophila*. Further, it has been correlated with decreased Aβ_42_ and Tau toxicity and improved mitochondrial function with Miro-overexpression in AD model flies. The results obtained from the current studies would be beneficial to find out further detailed molecular pathways associated with Miro and AD-associated genes.

**What has surprised you the most while conducting your research?**

As AD has been a devastating problem for humans, we were surprised to see the potential application/use of a tiny fruit fly *D. melanogaster* to find out the molecular details associated with AD. *Drosophila* model of AD mimics the AD related phenotypes and shows degenerated eyes, phototaxis impairment representing the sensory loss and climbing/locomotor defect representing motor dysfunction. Further, the gene associated with AD in humans has homologues in flies, and this motivated us to use this wonderful model organism for our studies.

**What, in your opinion, are some of the greatest achievements in your field and how has this influenced your research?**

Being a *Drosophila* researcher, I strongly believe that the investigation of different tools in *Drosophila* such as tissue targeting gene expression (UAS-GAL4), availability of different *Drosophila* mutants and reporter/UAS/knockdown fly lines are the greatest achievements in our field of research. The fly community is incredibly open and that helps a lot in facilitating *Drosophila* researchers to easily initiate their research.

**Figure:**
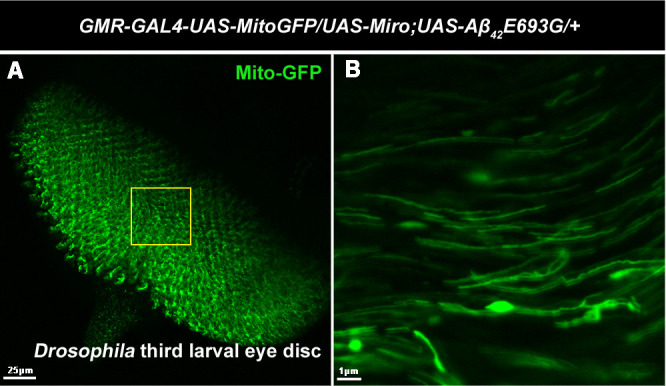
**The confocal image showing (A) GFP-tagged mitochondria (Mito-GFP) in third instar larval eye disc of *Miro*-overexpressing AD model flies (*GMR-GAL4-UAS-Mito-GFP/UAS-Miro;UAS-Aβ_42_E693G/+*).** (B) Magnified image of A showing that overexpression of *Miro* increased mitochondrial length (which represents mitochondrial fusion) in AD model flies.

**What changes do you think could improve the professional lives of early-career scientists?**

I believe that designing the proper hypothesis and research plan is key to success for early-career scientists. With strong basic knowledge and novel questions, early-career scientists can achieve and perform good research, which is beneficial to society.

**What's next for you?**

I am on the verge of my thesis submission and wish to get a postdoc position in a good research lab working in an area related to human diseases.
